# Pharmacokinetic Characteristics, Pharmacodynamic Effect and *In Vivo* Antiviral Efficacy of Liver-Targeted Interferon Alpha

**DOI:** 10.1371/journal.pone.0117847

**Published:** 2015-02-17

**Authors:** Daniel Rycroft, Jane Sosabowski, Edward Coulstock, Marie Davies, John Morrey, Sarah Friel, Fiona Kelly, Robert Hamatake, Milan Ovečka, Rob Prince, Laura Goodall, Armin Sepp, Adam Walker

**Affiliations:** 1 Biopharm Innovation Unit, Biopharm Research and Development, GlaxoSmithKline, Cambridge, United Kingdom; 2 Centre for Molecular Oncology, Barts Cancer Institute, Queen Mary University of London, London, United Kingdom; 3 Institute for Antiviral Research, Utah State University, Logan, Utah, United States of America; 4 Biopharm Translational Medicine, Biopharm R&D, GlaxoSmithKline, Stevenage, United Kingdom; 5 GlaxoSmithKline, Research Triangle Park, North Carolina, United States of America; 6 Biopharm Discovery, Biopharm R&D, GlaxoSmithKline, Stevenage, United Kingdom; Centro de Investigación en Medicina Aplicada (CIMA), SPAIN

## Abstract

Interferon alpha (IFNα) is used for the treatment of hepatitis B virus infection, and whilst efficacious, it is associated with multiple adverse events caused by systemic exposure to interferon. We therefore hypothesise that targeting IFN directly to the intended site of action in the liver would reduce exposure in blood and peripheral tissue and hence improve the safety and tolerability of IFNα therapy. Furthermore we investigated whether directing IFN to the reservoir of infection in the liver may improve antiviral efficacy by increasing local concentration in target organs and tissues. Our previous results show that the mIFNα2 fused to an ASGPR specific liver targeting antibody, DOM26h-196-61, results in a fusion protein which retains the activity of both fusion partners when measured *in vitro. In vivo* targeting of the liver by mIFNα2-DOM26h-196-61, hereafter referred to as targeted mIFNα2, was observed in microSPECT imaging studies in mice. In this study we show by pharmacokinetic analysis that antibody mediated liver-targeting results in increased uptake and exposure of targeted mIFNα2 in target tissues, and correspondingly reduced uptake and exposure in systemic circulation, clearance organs and non-target tissues. We also show that cytokine activity and antiviral activity of liver-targeted IFN is observed *in vivo*, but that, contrary to expectations, liver-targeting of mIFNα2 using ASGPR specific dAbs actually leads to a reduced pharmacodynamic effect in target organs and lower antiviral activity *in vivo* when compared to non-targeted mIFNα2-dAb fusions.

## Introduction

The current standard of care for hepatitis B virus (HBV) infection is treatment with pegylated IFN alpha [1, 2]. The potent anti-viral, anti-proliferative and immunomodulatory mechanisms of the type I interferons, a class of cytokines to which IFNα belongs, are well documented [3]. Whilst clearly efficacious, the systemic delivery of IFNα not only generates an anti-viral response in the liver, but also results in leukocyte activation in the blood leading to adverse responses to the therapy including cytokine release, flu-like symptoms and depression. These side-effects can be severe which leads to a significant proportion of patients discontinuing treatment [4, 5, 6].

The targeting of bioactive molecules to tissues is an attractive concept and in particular may offer multiple benefits in the treatment of HBV with IFNα. The perceived benefits are two-fold, namely increasing the local concentration of a therapeutic compound at the required site of action, potentially retaining efficacy with a reduced dose, and reducing undesired activity of a therapeutic in non-target tissues, potentially improving safety and tolerability. The application of this concept in multiple disease indications has been investigated using a wide range of methodologies, for example site-specific delivery of cytotoxic drugs for cancer therapy [7, 8], liposomal delivery of antigens in vaccine development [9] and the targeting of blood-brain barrier (BBB) receptors to facilitate transfer of biopharmaceuticals from the blood into the brain parenchyma [10].

Viral replication in HBV infection occurs predominantly in the liver. Asialoglycoprotein receptor (ASGPR) is a cell surface receptor expressed exclusively in hepatic parenchymal cells [11]. ASGPR is a C-type (calcium dependent) lectin composed of two transmembrane glycoprotein subunits, termed H1 and H2. The aglycosyl H1 and H2 subunits are approximately 35 and 33 kDa in size respectively, though purified ASGPR protein subunits are significantly larger due to post-translational modification. ASGPR mediates endocytosis of plasma glycoproteins that have exposed terminal galactose residues from which terminal sialic residues have been removed [12]. In addition, ASGPR has also been linked to the entry of HBV into hepatocytes [13]. Despite reports of potential extra hepatic expression in human kidney [14], thyroid [15] and activated T cells [16], ASGPR has been exploited in the targeting of therapeutic molecules to the liver. For example, ASGPR-targeted nanoparticles loaded with cytotoxic agents such as paclitaxel result in enhanced cell killing activity against ASGPR-positive cell lines when compared with free paclitaxel [17]. ASGPR-directed nanoparticles have also been used to deliver transgenes and antisense oligonucleotides to ASGPR-expressing primary hepatocytes and cell lines [18, 19]. *In vivo* radioiodinated copolymers with ASGPR binding activity accumulate in the liver following intravenous administration in rats [20]. In a study conducted by Peng *et al*., systemic delivery of the apoptin gene, which selectively induces apoptosis in malignant cells, linked to asialoglycoprotein resulted in specific delivery to ASGPR-positive HepG2 derived tumors xenografted in SCID mice and significant tumour regression. By contrast ASGPR-apoptin transgene conjugates were not able to induce tumour regression in non-hepatocyte derived A549 xenografted animals [21].

Compelling evidence for the potential application of ASGPR-mediated hepatic delivery in improving antiviral efficacy of type I interferons is provided in a study by Eto and Takahashi. Following enzymatic removal of terminal sialic acid residues from the N-linked oligosaccharide chain of human interferon beta (IFNβ), the investigators were able to demonstrate enhanced interferon signaling activity and inhibition of viral replication in HBV transfected HepG2 cells compared to the unmodified form of the protein [22]. This enhanced antiviral activity was presumably due to ASGPR binding, as it could be partially inhibited by natural ASGPR ligands such as asialofetuin. Significantly enhanced *in vivo* antiviral efficacy of murine asialo-IFNβ, compared with that of the unmodified protein, was also shown in HBV transfected BALB/c athymic nude mice.

The small size of dAbs (11–15kDa) coupled with their high affinity for their respective antigen can help preserve the activity of fusion partners, which makes their use attractive [23, 24, 25]. We have previously shown that IFNα-ASGPR dAb fusion proteins can be expressed in mammalian cells whilst retaining the *in vitro* activity of both fusion partners. Furthermore, using SPECT imaging we have shown that the fusion protein targeted mIFNα2 specifically targets the liver *in vivo* [26]. In this study we show that targeted mIFNα2 exposure is increased in target organs and reduced in systemic circulation and non-target tissues. Whilst targeted mIFNα2 induces IFN-responsive gene expression and elicits antiviral effects *in vivo*, these effects are lower in comparison to non-targeted mIFNα-dAb fusion proteins, suggesting that the targeting method has more utility in reducing exposure in non-target tissues and organs than increasing efficacy as a direct consequence of increased local concentration in target tissues and organs.

## Materials and Methods

### Production and characterisation of mIFNα2-dAb fusion proteins and PEGylated mIFNα2

Fusion proteins and PEGylated mIFNα2 were produced and characterised as described previously [26] with the following additional steps to produced PEGylated mIFNα2; 16.75 fold molar excess of 40kDa Branched PEG NHS-ester (TOF Sunbright) was added to mIFNα2 in PBS (Sigma Aldrich). Reaction was incubated at room temperature for 2hrs before purification using ion exchange chromatography using a 1ml Resource S column (GE Healthcare).

### Conjugation of mIFNα2-dAb fusion proteins with NHS-DOTA

Fusion proteins were dialysed into 25 mM Na Acetate solution, pH 8 (Sigma Aldrich) using Maxi GeBAflex dialysis tubes with a 3.5 kDa molecular weight cut off (Gene Bio-Application Ltd.). NHS-DOTA (Macrocyclics Inc.) was then added in a 4-fold molar excess and reacted overnight at room temperature. Conjugation solutions were then applied to Protein A columns (GE Healthcare) equilibrated in Chelex 100 (Bio-Rad Ltd.) treated PBS, pH 7.4 (PAA Laboratories GmbH), washed with Chelex treated PBS and eluted in 0.5 ml fractions of 0.1 M glycine/HCl, pH 2 (Sigma Aldrich), into tubes containing ammonium acetate (final concentration/pH of fractions was 0.46 M ammonium acetate, pH 5). Conjugation efficiency and activity were determined as described previously [26].

### Radiolabelling and radiochemical analysis of DOTA conjugated mIFNα2-dAb fusion proteins

All radiolabelling was carried out 4, 3 or 2 days prior to the ^111^InCl_3_ reference date when the radioactivity concentration was approximately 1, 0.83 or 0.65 MBq/μl respectively. The general radiolabelling protocol was as follows; to a low protein binding 1.5 ml polypropylene tube (Nunc) was added 40–60 μl (26–50 MBq) of ^111^InCl_3_ (Covidien) in 0.05 M HCl (Sigma Aldrich), 8–12 μl (1/5th the volume) of 1 M ammonium acetate (Sigma Aldrich), pH 4.5–5.5 (or in the case of mIFNα2-DOM26h-196-61, 120 μl 0.1 M MES, pH 5.1 (Sigma Aldrich)) and 12.5–92.5 μg of protein. The solution was heated to 40°C for 1.5–2.5 h and quenched with 0.1 M EDTA solution (Norwich NHS Trust) using 1/20th reaction volume. Radiochemical purity was determined using size exclusion HPLC and thin layer chromatography (TLC) analysis after which the reaction mixture was diluted with PBS or PBS/ 0.1% BSA (Sigma Aldrich) followed by filtration through a 0.22 μm filter.

### Pharmacokinetic studies in CD-1 mice

All animal studies were ethically reviewed and carried out in accordance with Animals (Scientific Procedures) Act 1986 and the GlaxoSmithKline Policy on the Care, Welfare and Treatment of Animals.

All studies were conducted in accordance with the GlaxoSmithKline Policy on the Care, Welfare and Treatment of Laboratory Animals and were reviewed by the Institutional Animal Care and Use Committee either at GlaxoSmithKline or by the ethical review process at the institution where the work was performed. The Institutional Animal Care and Use Committee specifically approved this study.

Fusion proteins were administered as a single intravenous dose at 5mg/kg into a group of male CD-1 mice via the caudal vein, or as a single subcutaneous dose at 5mg/kg into a group of male CD-1 mice. At a range of time points up to 120 hours post dose. Blood from 3 mice of each group was taken as either small volume tail bleeds or as terminal bleeds following sacrifice. Blood samples were allowed to clot at ambient temperature, before samples were centrifuged at approximately 2000g for 10 minutes to prepare serum. Serum was stored frozen until required.

### Analysis of mIFNα2-dAb fusion proteins using antibody capture and detection

The concentrations of any mIFNα2-V_H_ dAb fusion in mouse serum samples was determined using an MSD assay. Briefly, 96-well standard bind MSD plates (Mesoscale Discovery) were coated overnight with a rat anti-mouse IFNα mAb (R&D Systems). The following day, plates were washed with PBS/0.1% Tween-20. Wells were then blocked with assay buffer (5% BSA in PBS containing 1% tween-20).

Standard samples and study samples were added at a range of dilutions in duplicate. Samples were diluted in assay buffer containing an appropriate amount of control matrix to match matrix concentrations across the plate. The appropriate mIFNα2-V_H_ dAb fusion was added to each plate as triplicate standard curves at a range of known concentrations in assay buffer containing an appropriate amount of control matrix.

After washing, bound mIFNα2-V_H_ dAb fusion was detected with MSD sulfo-tagged mouse anti-V_H_ mAb (in-house reagent, clone M2.3G10.1G06, prepared according to MSD protocols). Plates were read on a SECTOR 6000 MSD imager.

### Pharmacokinetic studies in BALB/c mice

Radiolabelled mIFNα2-dAb fusions were administered as a single intravenous dose at 20μg/kg into the tail vein of a group of male BALB/C mice. At a range of time points up to 96 hours post dose terminal blood samples from 3 mice of each group were taken. Following sacrifice, the kidneys, liver and a suitable amount of muscle was extracted. Radioactivity levels were then quantified in all sample types using gamma counting.

### Analysis of pharmacokinetic data

Final assay results were fitted in WinNonLin by NCA according to standard methods. The mean PK was plotted using Graphpad Prism version 6. Derived PK parameters were obtained from the NCA fit.

### Quantitative PCR Analysis of Interferon Inducible Gene Expression

Purification of total RNA >200 nucleotides (excluding miRNA) from blood was carried out using RNeasy protect animal blood kit (Qiagen). 200 ng of RNA was arrayed in triplicate in 96 well plates on ice. Reverse transcription reactions were set up in triplicate converting using a high capacity cDNA conversion kit (Applied Biosystems) following manufacturer's instructions. On completion of cDNA conversion samples were diluted to 5ng/ul of input RNA and arrayed in 384 well plate formats at 10 ng/well and stored at frozen until required.

Liver tissue samples were placed in RNALater (Qiagen) as per manufacturer’s instructions. Livers were then homogenised in Trizol (LifeTechnologies) at a ratio of 100 mg tissue per ml liquid. A 750 μl aliquot was made of each sample and stored frozen until processed.

150 μl of chloroform was then added to thawed homogenates, before mixing for 5 minutes at room temperature. Homogenates were then centrifuged for 15 minutes before transfer of aqueous phase of the sample to ethanol to provide appropriate binding conditions. Samples were then applied to RNeasy 96 well plates (Qiagen) to allow total RNA binding. Contaminants were washed away using supplied buffers before application of DNase to allow digestion of remaining DNA. DNase was then removed by washing plates with supplied buffers. The RNeasy RNA membrane was then dried to remove any ethanol. High-quality RNA was then eluted in 70μl water.

2.5 μg of RNA was arrayed in triplicate in 96 well plates on ice. Reverse transcription reactions were set up in triplicate using a high capacity cDNA conversion kit (Applied Biosytems) following manufacturer's instructions. On completion of cDNA conversion samples diluted to 10 ng/ul of input RNA and arrayed in 384 well plate format at 20 ng/well and stored at frozen until required.

TaqMan reaction plates were then set up by addition of universal PCR master mix (Applied Biosystems), adding 8 μl of master mix to the 2ul of cDNA template previously plated into the 384-well plates.1 μl each primer and 2 μl of water was added per well, giving final concentration of 900nM forward primer, 900nM reverse primer and 100nM probe. Plates were cycled on an ABI7900 HT TaqMan machine (Applied Biosystems) using the following cycling conditions; 50oC for 2min, 95oC for 10 min, followed by 40 cycles of 95oC for 15sec and 60oC for 1min. All data analysis was performed in Array Studio v3.5. Data points were excluded if Ct's were greater than 35 or less than 10. Technical triplicates were assessed for variability using the following method; if the range of Ct's for a set of triplicates is greater than 1 Ct, the raw SDS traces are examined for evidence of an inefficient reaction. If a replicate is an outlier greater than 1 Ct and shows evidence of an inefficient reaction then it was excluded from the study. Ct values were converted into abundances (copies/50ng RNA for liver and /25ng RNA for blood) using genomic standards run on the same plate.

Abundances for each gene were normalised using the scores from first principal component from a principal component analysis on selected invariant genes GAPDH, ACTB and PPIB. The normalising constant was fitted in the final model as a covariate so that the data was normalised and analysed simultaneously.

### Determination of antiviral efficacy in HBV transgenic mouse model

Transgenic HBV mice originally obtained from Dr. Frank Chisari (Scripps Research Institute, LaJolla, CA) were used in this study. Homozygous animals were raised in the Biosafety Level 3 area of the USU Laboratory Animal Research Center (LARC). The animals were derived from founder 1.3.32. Female and male mice 12–16 weeks old were assigned randomly to treatment groups. Animals were treated once intravenously with doses of 2, 20, and 200 μg/kg of targeted mIFNα2 or non-targeted mIFNα2, with 1.2, 12, or 120 μg/kg of PEGylated mIFNα2, or with a sterile sodium acetate (pH 5.5) vehicle. Ten animals were included in each group. At 24 hr after injection, the mice were necropsied to obtain samples for liver HBV DNA using qPCR and Southern blot hybridization.

Liver HBV DNA was analysed by Southern blot hybridization and by real-time PCR. The procedures for preparation of liver tissue, Southern blot hybridization and PCR are described previously [27].

## Results

### Pharmacokinetic analysis of mIFNα2-dAb fusion proteins following intravenous administration

Following a single intravenous bolus administration of mIFNα2-V_H_D2 (hereafter referred to as non-targeted mIFNα2) and targeted mIFNα2 fusion proteins in male CD-1 mice, serum levels were determined using an MSD based assay. This assay format utilises capture via murine IFNα specific rabbit polyclonal antisera and detection via sulfo-tagged anti-V_H_ mouse monoclonal antibody and is, therefore, designed to measure the level of intact fusion protein in biological matrices.

**Fig 1:**
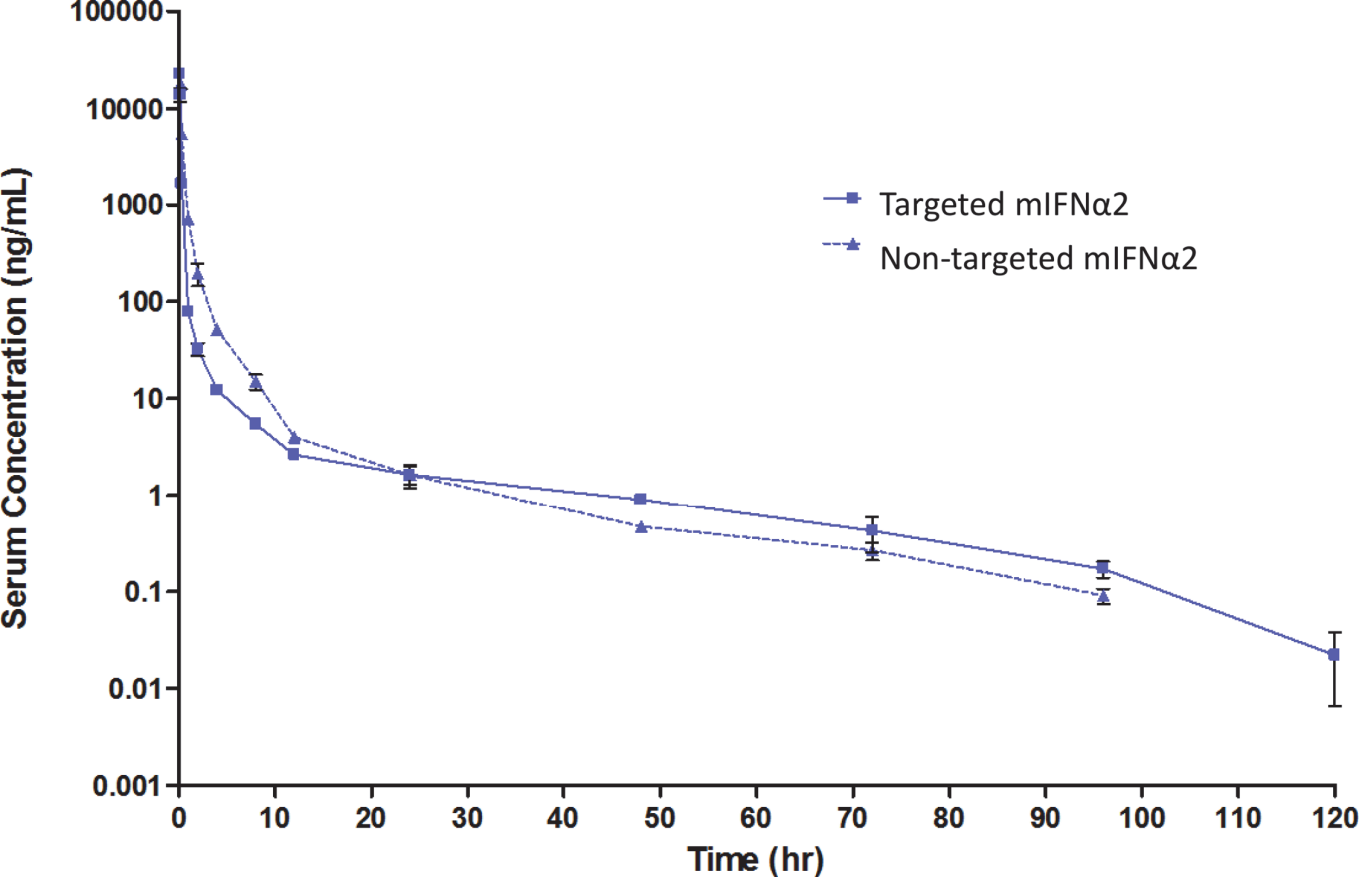
Pharmacokinetic analysis of mIFNα2-dAb fusions in serum following intravenous administration. Targeted mIFNα2 (solid line) and non-targeted mIFNα2 isotype control dAb (dotted line) were administered at 5mg/kg via intravenous injection. Compound levels were analysed in serum by mIFNα2 specific antibody capture and dAb specific detection. Data shown are mean n = 3 animals. Error bars represent s.e.m. Pharmacokinetic parameters are shown in Table 1.

Fig. 1 shows the pharmacokinetic profiles of non-targeted mIFNα2 and targeted mIFNα2 in the serum of male CD-1 mice after a 5 mg/kg intravenous dose. Table 1 shows a summary of the derived key parameters from non-compartmental analysis of the data. Some individual animals were excluded from the plot shown and were not included in the analysis as they were suspected outliers.

**Table 1:** Pharmacokinetics of non-targeted mIFNα2 and targeted mIFNα2 in mouse serum after a single 5 mg/kg intravenous dose.

Molecule	T ½ (hr)	Tmax (hr)	Cmax (ng/mL)	SE of Cmax	AUC_(0-∞)_ (hr*ng/mL)	Vz (mL/kg)	Cl (mL/kg/hr)	MRT (hr)
non-targeted mIFNα2	16.2	0.17	17,743.0	1,104.9	11,659.7	9,996.1	428.8	0.5
targeted mIFNα2	17.1	0.08	22,740.0	50.9	6,028.0	20,432.5	829.5	0.7

Non-targeted mIFNα2 had a terminal half-life of 16.2 hours, clearance of 428 mL/kg/hr, a volume of distribution of 9,996 mL/kg and an AUC_(0-∞)_ of 11,660 hr*ng/mL. Targeted mIFNα2 had a terminal half-life of 17.1 hours, clearance of 829 mL/kg/hr, a volume of distribution of 20,432 mL/kg and an AUC_(0-∞)_ of 6,028 hr*ng/mL.

### Pharmacokinetic analysis of mIFNα2-dAb fusion proteins following subcutaneous administration

Following a single subcutaneous administration of non-targeted mIFNα2 and targeted mIFNα2 fusion proteins in male CD-1 mice, serum levels were determined using the MSD assay described above, in order to determine whether differences in the pharmacokinetic parameters of targeted and non-targeted mIFNα2-dAb fusion proteins would be observed when using a route of administration other than intravenous injection.

**Fig 2:**
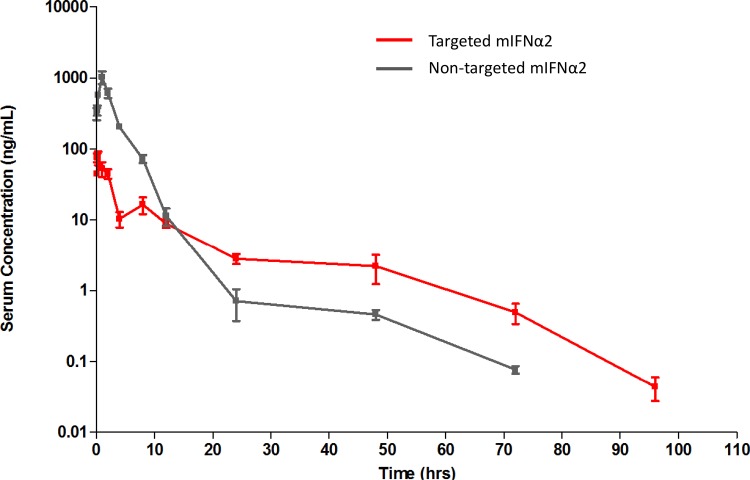
Pharmacokinetic analysis of mIFNα2-dAb fusions in serum following subcutaneous administration. Targeted mIFNα2 (red line) and non-targeted mIFNα2 isotype control dAb (grey line) were administered at 5mg/kg via subcutaneous injection. Compound levels were analysed in serum by mIFNα2 specific antibody capture and dAb specific detection. Data shown are mean n = 3 animals. Error bars represent s.e.m. Pharmacokinetic parameters are shown in Table 2.

Fig. 2 shows the pharmacokinetic profiles of non-targeted mIFNα2 and targeted mIFNα2 in the serum of male CD-1 mice after a 5 mg/kg subcutaneous dose. Table 2 shows a summary of the derived key PK parameters from the non-compartmental analysis of the data.

**Table 2:** Pharmacokinetics of non-targeted mIFNα2 and targeted mIFNα2 in mouse serum after a single 5 mg/kg subcutaneous dose.

Molecule	T ½ (hr)	Tmax (hr)	Cmax (ng/mL)	SE of Cmax	AUC_(0-∞)_ (hr*ng/mL)	Vz_F (mL/kg)	Cl_F (mL/kg/hr)	MRT (hr)	F (%)
non-targeted mIFNα2	15.0	1	1,026.4	207.6	3,103.9	34,775.9	1,610.9	3.1	26.6
targeted mIFNα2	8.5	0.16	75.5	10.9	440.8	138,786.6	11,342.8	14.6	7.3

In serum targeted mIFNα2 had a terminal half-life of 8.5 hours, Tmax of 0.17 hours, clearance (of fraction absorbed) of 11,343 mL/hr/kg, a volume of distribution (of fraction absorbed) of 138,787 mL/kg and an AUC_(0-∞)_ of 440 hr*ng/mL. non-targeted mIFNα2 had a terminal half-life of 15.0 hours, Tmax of 1 hour, clearance (of fraction absorbed) of 1,611 mL/hr/kg, a volume of distribution (of fraction absorbed) of 34,776 mL/kg and an AUC_(0-∞)_ of 3,104 hr*ng/mL. Using the an AUC_(0-∞)_ calculated here and from those determined in Fig. 1 (see Table 1) it was possible to determine the systemic bioavailability of each molecule. This was determined to be 7.3% for targeted mIFNα2 and 26.6% for non-targeted mIFNα2.

### Pharmacokinetic analysis of ^111^In-DOTA-non-targeted mIFNα2 and ^111^In-DOTA-targeted mIFNα2 in blood following intravenous administration

The studies described above to determine pharmacokinetic parameters of intact fusion proteins in serum were carried out at doses much higher than the anticipated clinical dose for IFN therapy in humans. This was a requirement due to the sensitivity of the assay format. Therefore in order to investigate the kinetics of targeted mIFNα2 and non-targeted mIFNα2 at a more clinically relevant dose, a study was carried out to measure the concentrations of DOTA conjugated non-targeted mIFNα2 and targeted mIFNα2, labelled with ^111^In, in whole blood. This would in all likelihood overcome the incompatibility between low dose administration of compound and the known sensitivity of the assay described in Figs. 1 and 2. ^111^In-DOTA-non-targeted mIFNα2 and ^111^In-DOTA- targeted mIFNα2 were administered via a single intravenous injection at 20 μg/kg into the tail vein of male BALB/c mice. At a range of time points, terminal blood samples were taken and radioactivity levels quantified in a gamma counter.

**Fig 3:**
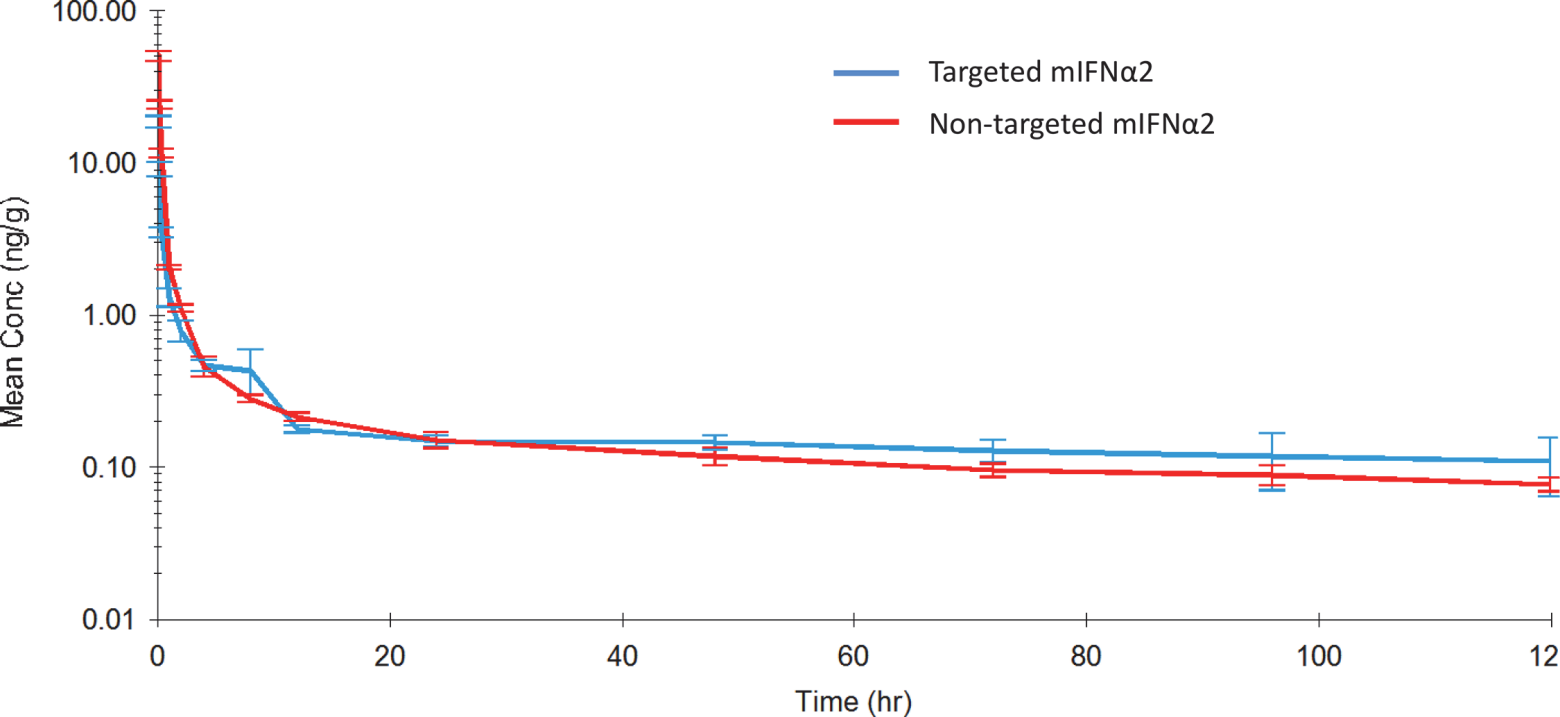
Pharmacokinetic analysis of ^111^In-DOTA-mIFNα2-dAb fusions in blood following intravenous administration. Targeted mIFNα2 (blue line) and non-targeted mIFNα2 isotype control dAb (red line) were administered at 20μg/kg via intravenous injection. Radioactivity levels were analysed in blood by gamma counting. Data shown are mean n = 3 animals. Error bars represent s.e.m. Pharmacokinetic parameters are shown in Table 3.

Fig. 3 shows the pharmacokinetic profiles of ^111^In-DOTA-non-targeted mIFNα2 and ^111^In-DOTA-targeted mIFNα2 in mice after a 20 μg/kg intravenous dose. Table 3 shows a summary of the derived key parameters from the non-compartmental analysis of the data.

**Table 3:** Pharmacokinetics of ^111^In-DOTA-non-targeted mIFNα2 and ^111^In-DOTA- targeted mIFNα2 in mouse blood after a single 20 μg/kg intravenous dose.

Molecule	T ½ (hr)	Tmax (hr)	Cmax (ng/mL)	SE of Cmax	AUC_(0-∞)_ (hr*ng/mL)	Vz (mL/kg)	Cl (mL/kg/hr)	MRT (hr)	AUC_(0–12)_ (hr*ng/mL)	AUC_(0–24)_ (hr*ng/mL)
^111^In-DOTA-non-targeted mIFNα2	80.4	0.083	50.2	3.6	42.7	54,331.0	468.7	61.9	22.4	24.6
^111^In-DOTA-targeted mIFNα2	171.7	0.083	18.6	1.6	52.4	94,650.2	382.0	204.3	11.4	13.3

In blood, ^111^In-DOTA-targeted mIFNα2 had a terminal half-life of 171 hours, clearance of 382 mL/hr/kg, a volume of distribution of 94,650 mL/kg, an AUC_(0-∞)_ of 52.4 hr*ng/mL, an AUC_(0–12)_ of 11.4 hr*ng/mL and an AUC_(0–24)_ of 13.3 hr*ng/mL. ^111^In-DOTA-non-targeted mIFNα2 had a terminal half-life of 80.4 hours, clearance of 468 mL/hr/kg, a volume of distribution of 54,331 mL/kg and an AUC_(0-∞)_ of 42.7 hr*ng/mL, an AUC_(0–12)_ of 22.4 hr*ng/mL and an AUC_(0–24)_ of 24.6 hr*ng/mL.

### Pharmacokinetic analysis of ^111^In-DOTA-non-targeted mIFNα2 and ^111^In-DOTA- targeted mIFNα2 in liver following intravenous administration

In order to determine the pharmacokinetic parameters of targeted mIFNα2 and non-targeted mIFNα2 in the target organ, livers of mice in Fig. 3 were collected and radioactivity levels quantified in a gamma counter. This method, in addition to overcoming assay sensitivity issues described above, would also overcome potential variability introduced by the requirement to extract compound prior to detection using antibody based methods. The results obtained in tissue using ^111^In labelled compounds would be independent of the extraction efficiency of the processes used during the preparation of homogenate supernatants, and therefore would perhaps be a more appropriate way of investigating tissue distribution over time.

**Fig 4:**
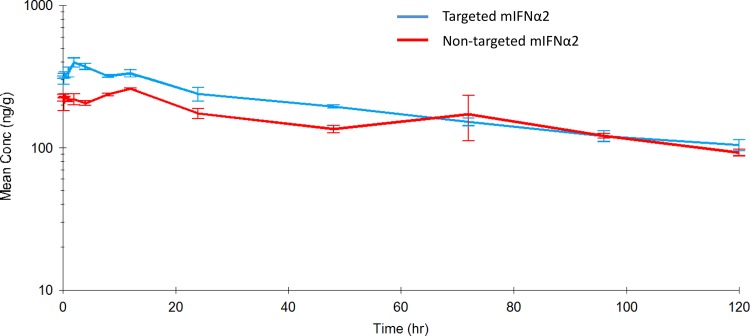
Pharmacokinetic analysis of ^111^In-DOTA-mIFNα2-dAb fusions in liver following intravenous administration. Targeted mIFNα2 (blue line) and non-targeted mIFNα2 isotype control dAb (red line) were administered at 20μg/kg via intravenous injection. Radioactivity levels were analysed in liver by gamma counting. Data shown are mean n = 3 animals. Error bars represent s.e.m. Pharmacokinetic parameters are shown in Table 4.

Fig. 4 shows the pharmacokinetic profiles of ^111^In-DOTA-non-targeted mIFNα2 and ^111^In-DOTA- targeted mIFNα2 in the liver of male BALB/c mice after a 20 μg/kg intravenous dose. Table 4 shows a summary of the derived key parameters from the non-compartmental analysis of the data.

**Table 4:** Pharmacokinetics of ^111^In-DOTA-non-targeted mIFNα2 and ^111^In-DOTA- targeted mIFNα2 in mouse liver after a single 20 μg/kg intravenous dose.

Molecule	T ½ (hr)	Tmax (hr)	Cmax (ng/g)	SE of Cmax	AUC_(0-∞)_ (hr*ng/g)	AUC_(0–12)_ (hr*ng/g)	AUC_(0–24)_ (hr*ng/g)
^111^In-DOTA-non-targeted mIFNα2	92.9	12	259.1	3.3	31,945.3	2,735.8	5,333.8
^111^In-DOTA-targeted mIFNα2	67.9	2	397.6	30.1	32,474.0	4,159.0	7,592.6

In liver, ^111^In-DOTA- targeted mIFNα2 had a terminal half-life of 67.9 hours, Cmax of 397 ng/g, Tmax of 2 hours, an AUC_(0-∞)_ of 32,474 hr*ng/g, an AUC_(0–12)_ of 4,159 hr*ng/mL and an AUC_(0–24)_ of 7,592 hr*ng/mL. ^111^In-DOTA-non-targeted mIFNα2 had a terminal half-life of 92.9 hours, Cmax of 259 ng/g, Tmax of 12 hours, an AUC_(0-∞)_ of 31,945 hr*ng/g, an AUC_(0–12)_ of 2,735 hr*ng/mL and an AUC_(0–24)_ of 5,333 hr*ng/mL.

### Pharmacokinetic analysis of ^111^In-DOTA-non-targeted mIFNα2 and ^111^In-DOTA-targeted mIFNα2 in kidney following intravenous administration

In order to determine the pharmacokinetic parameters of targeted mIFNα2 and non-targeted mIFNα2 in non-target organs, kidneys of mice in Fig. 3 were removed and radioactivity levels quantified in a gamma counter. As the primary route of clearance of ^111^In-DOTA-mIFNα2-dAb fusion proteins is likely to be via the kidney, analysing this tissue as an example of non-target organ/tissue would also allow us to further investigate differences in the clearance of these two molecules *in vivo*.

**Fig 5:**
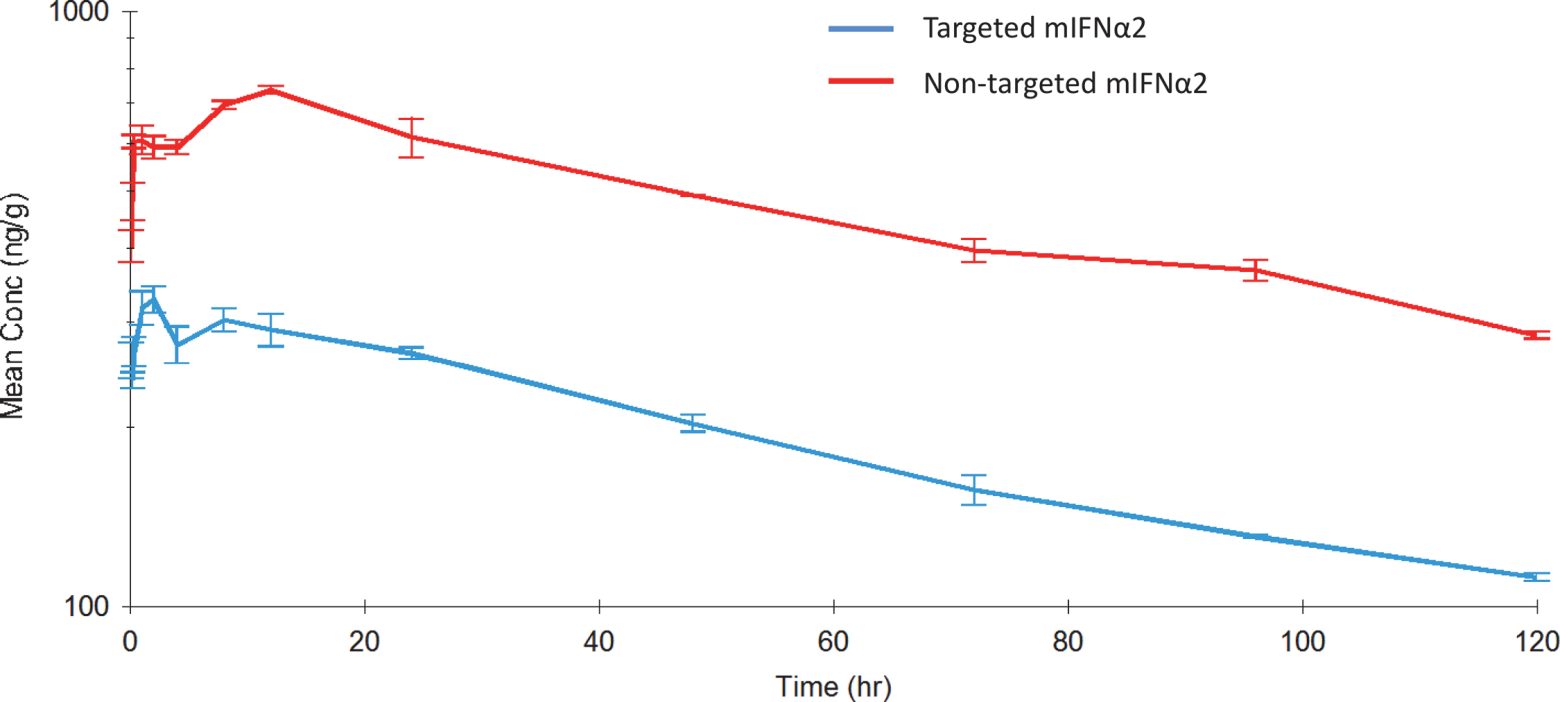
Pharmacokinetic analysis of ^111^In-DOTA-mIFNα2-dAb fusions in kidney following intravenous administration. Targeted mIFNα2 (blue line) and non-targeted mIFNα2 isotype control dAb (red line) were administered at 20μg/kg via intravenous injection. Radioactivity levels were analysed in kidney by gamma counting. Data shown are mean n = 3 animals. Error bars represent s.e.m. Pharmacokinetic parameters are shown in Table 5.

Fig. 5 shows the pharmacokinetic profiles of ^111^In-DOTA-non-targeted mIFNα2 and ^111^In-DOTA-targeted mIFNα2 in the kidney of male BALB/c mice after a 20 μg/kg intravenous dose. Table 5 shows a summary of the derived key parameters from the non-compartmental analysis of the data.

**Table 5:** Pharmacokinetics of ^111^In-DOTA-non-targeted mIFNα2 and ^111^In-DOTA- targeted mIFNα2 in mouse kidney after a single 20 μg/kg intravenous dose.

Molecule	T ½ (hr)	Tmax (hr)	Cmax (ng/g)	SE of Cmax	AUC_(0-∞)_ (hr*ng/g)	AUC_(0–12)_ (hr*ng/g)	AUC_(0–24)_ (hr*ng/g)
^111^In-DOTA-non-targeted mIFNα2	84.1	12	736.1	11.2	91,128.6	7,776.5	15,874.4
^111^In-DOTA-targeted mIFNα2	75.4	2	327.8	16.9	34,795.3	3,551.3	6,899.6

In kidney, ^111^In-DOTA-targeted mIFNα2 had a terminal half-life of 75.4 hours, Cmax of 327 ng/g, Tmax of 2 hours, an AUC_(0-∞)_ of 34,795 hr*ng/g, an AUC_(0–12)_ of 3,551 hr*ng/mL and an AUC_(0–24)_ of 6,899 hr*ng/mL. ^111^In-DOTA-non-targeted mIFNα2 had a terminal half-life of 84.1 hours, Cmax of 736 ng/g, Tmax of 12 hours, an AUC_(0-∞)_, an AUC_(0–12)_ of 7,776 hr*ng/mL and an AUC_(0–24)_ of 15,874 hr*ng/mL of 91,128 hr*ng/g.

### Systemic pharmacodynamic effect of mIFNα2-dAb fusion proteins following intravenous administration

In order to determine the *in vivo* pharmacodynamic effect of targeted mIFNα2 and non-targeted mIFNα2 in the systemic circulation we measured by quantitative PCR analysis the expression levels in blood of a panel of interferon inducible genes following intravenous administration of targeted mIFNα2 and non-targeted mIFNα2 at 2 μg/kg and 20 μg/kg. The interferon inducible genes analysed for expression were OAS1, OAS2, OAS3, ADAR, GBP1, CXCL10, IFIT1, EIF2AK2, and MX2. Only the data for EIF2AK2 are shown, but the gene expression pattern was broadly similar in the case of all interferon-inducible genes analysed.

A panel of non-inducible selected invariant genes genes was also analysed. Levels of ACTB, PPIB, and GAPDH were measured. The effect on gene expression was similar with both fusion proteins in the case of all 3 genes, though only the GAPDH analysis is shown.

Gene expression levels were also analysed in control animals administered with vehicle only.

**Fig 6:**
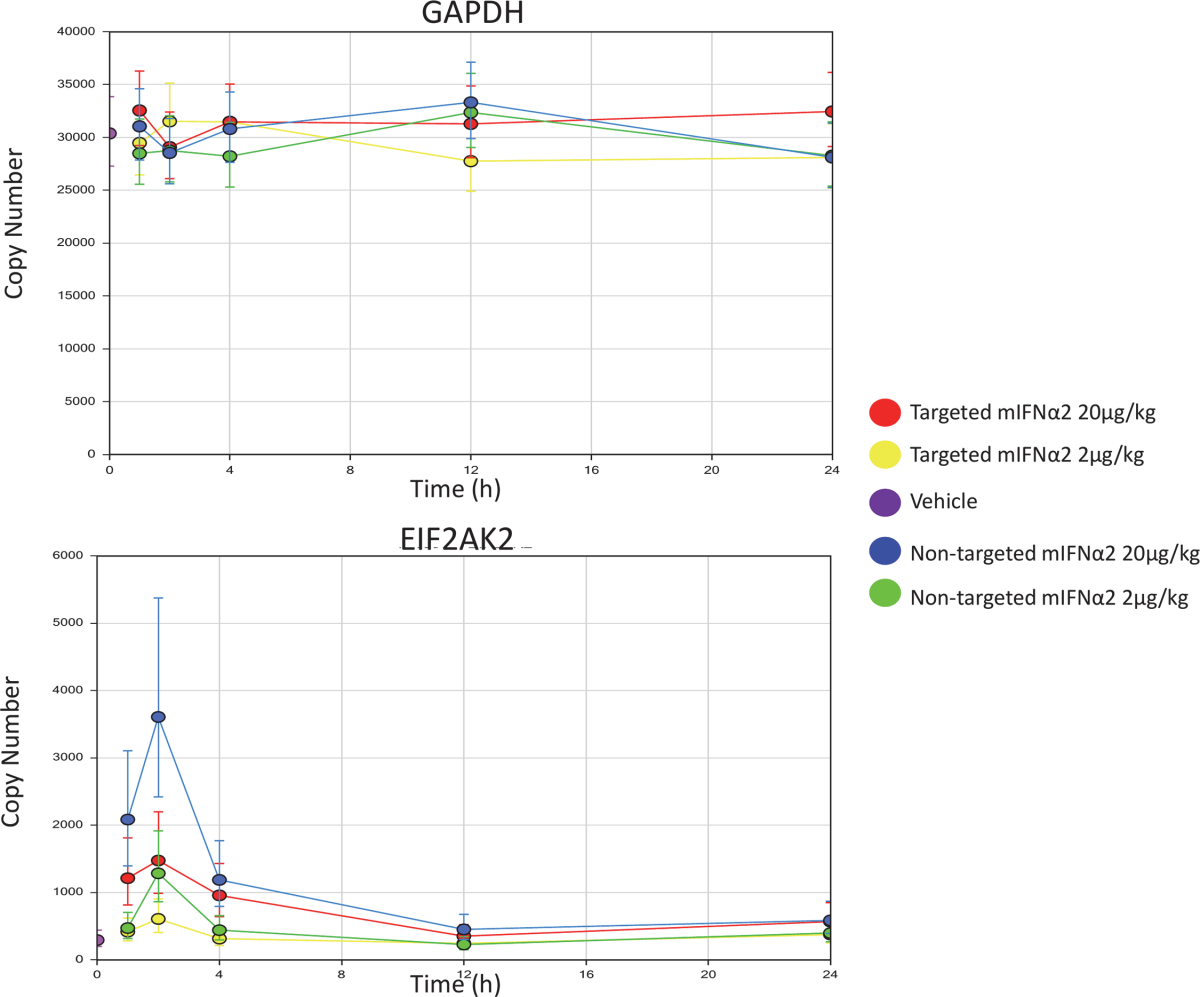
Analysis of IFN inducible gene expression in blood by mIFNα2-dAb fusions. Targeted mIFNα2 and non-targeted mIFNα2 were administered at 2μg/kg and 20μg/kg via intravenous injection. Vehicle control was also included. Levels of invariant and IFN inducible gene expression were analysed by TaqMan. Data shown are mean n = 4 animals. Error bars represent 95% CI.

Fig. 6 shows that interferon inducible gene expression is observed in blood following intravenous administration of targeted mIFNα2 and non-targeted mIFNα2. The level of interferon inducible gene expression is proportional to the dose of targeted mIFNα2 and non-targeted mIFNα2 administered, with a greater level of gene expression observed at the 20 μg/kg dose compared to the 2 μg/kg dose in the case of both fusion proteins.

Maximum levels of gene expression are observed at 1–2 hours following intravenous administration of both targeted mIFNα2 and non-targeted mIFNα2, though the levels of gene expression induced by non-targeted mIFNα2 appear to be higher at all time points up to 12 hours when compared with the levels induced by targeted mIFNα2. These data are consistent with the pharmacokinetic analysis of ^111^In-DOTA-non-targeted mIFNα2 and ^111^In-DOTA-targeted mIFNα2 described above, which shows that exposure of ^111^In-DOTA-non-targeted mIFNα2 in blood is higher compared to that of ^111^In-DOTA-targeted mIFNα2. By contrast no effect on GAPDH expression was observed following intravenous administration of either targeted mIFNα2 or non-targeted mIFNα2.

### Pharmacodynamic effect of mIFNα2-dAb fusion proteins in liver following intravenous administration

In order to determine the *in vivo* pharmacodynamic effect of targeted mIFNα2 and non-targeted mIFNα2 in the target tissue we measured the expression levels in liver of the same panel of interferon inducible genes described above following intravenous administration of targeted mIFNα2 and non-targeted mIFNα2 at 2 μg/kg and 20 μg/kg. Only the data for EIF2AK2 are shown, but the gene expression pattern was broadly similar in the case of all interferon-inducible genes analysed.

The same panel of non-inducible ‘house-keeping’ genes was also analysed and the effect on gene expression similar with both fusion proteins in the case of all 3 genes, though only the GAPDH analysis is shown.

Gene expression levels were also analysed in control animals administered with vehicle only.

**Fig 7:**
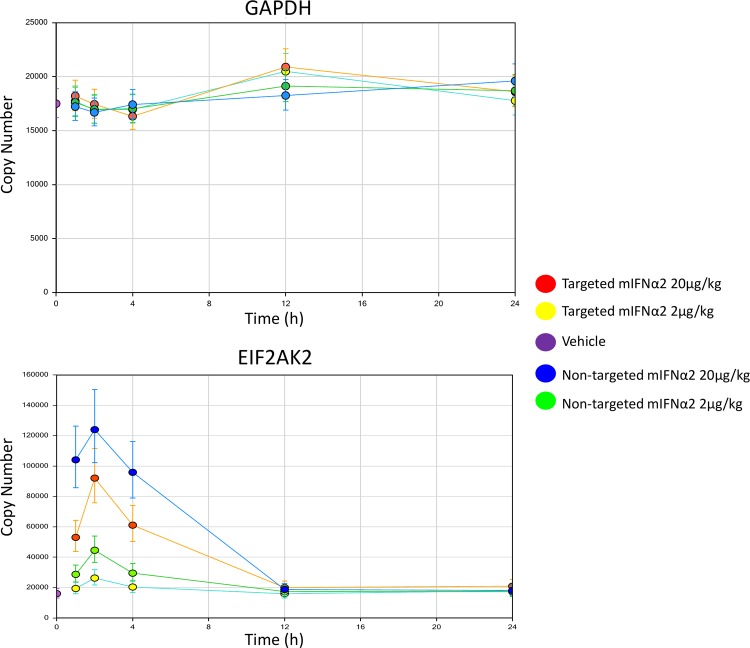
Analysis of IFN inducible gene expression in liver by mIFNα2-dAb fusions. Targeted mIFNα2 and non-targeted mIFNα2 were administered at 2μg/kg and 20μg/kg via intravenous injection. Vehicle control was also included. Levels of invariant and IFN inducible gene expression were analysed by TaqMan. Data shown are mean n = 4 animals. Error bars represent 95% CI.

Fig. 7 shows that interferon inducible gene expression is observed in liver following intravenous administration of targeted mIFNα2 and non-targeted mIFNα2. The level of interferon inducible gene expression is proportional to the dose of targeted mIFNα2 and non-targeted mIFNα2 administered, with a greater level of gene expression observed at the 20 μg/kg dose compared to the 2 μg/kg dose in the case of both fusion proteins.

Maximum levels of gene expression are observed at 1–2 hours following intravenous administration of both targeted mIFNα2 and non-targeted mIFNα2, though the levels of gene expression induced by non-targeted mIFNα2 appear to be higher at all time points up to 12 hours when compared with the levels induced by targeted mIFNα2. This data is in contrast to the pharmacokinetic analysis of ^111^In-DOTA-non-targeted mIFNα2 and ^111^In-DOTA- targeted mIFNα2 described above, which shows that exposure of ^111^In-DOTA-non-targeted mIFNα2 in liver is lower compared to that of ^111^In-DOTA- targeted mIFNα2. No effect on GAPDH expression is observed following intravenous administration of either targeted mIFNα2 or non-targeted mIFNα2.

### *In vivo* antiviral efficacy of non-targeted mIFNα2, targeted mIFNα2 and PEGylated mIFNα2 in HBV transgenic mice

In order to determine the *in vivo* antiviral efficacy of targeted mIFNα2 and non-targeted mIFNα2, compounds were administered intravenously at 3 doses in transgenic mice, which stably express human hepatitis B virus particles. Efficacy was determined by measuring ability of mIFNα2-dAb fusions to inhibit viral DNA replication, and a PEGylated mIFNα2 was included as a murine version of clinically relevant compound.

**Fig 8:**
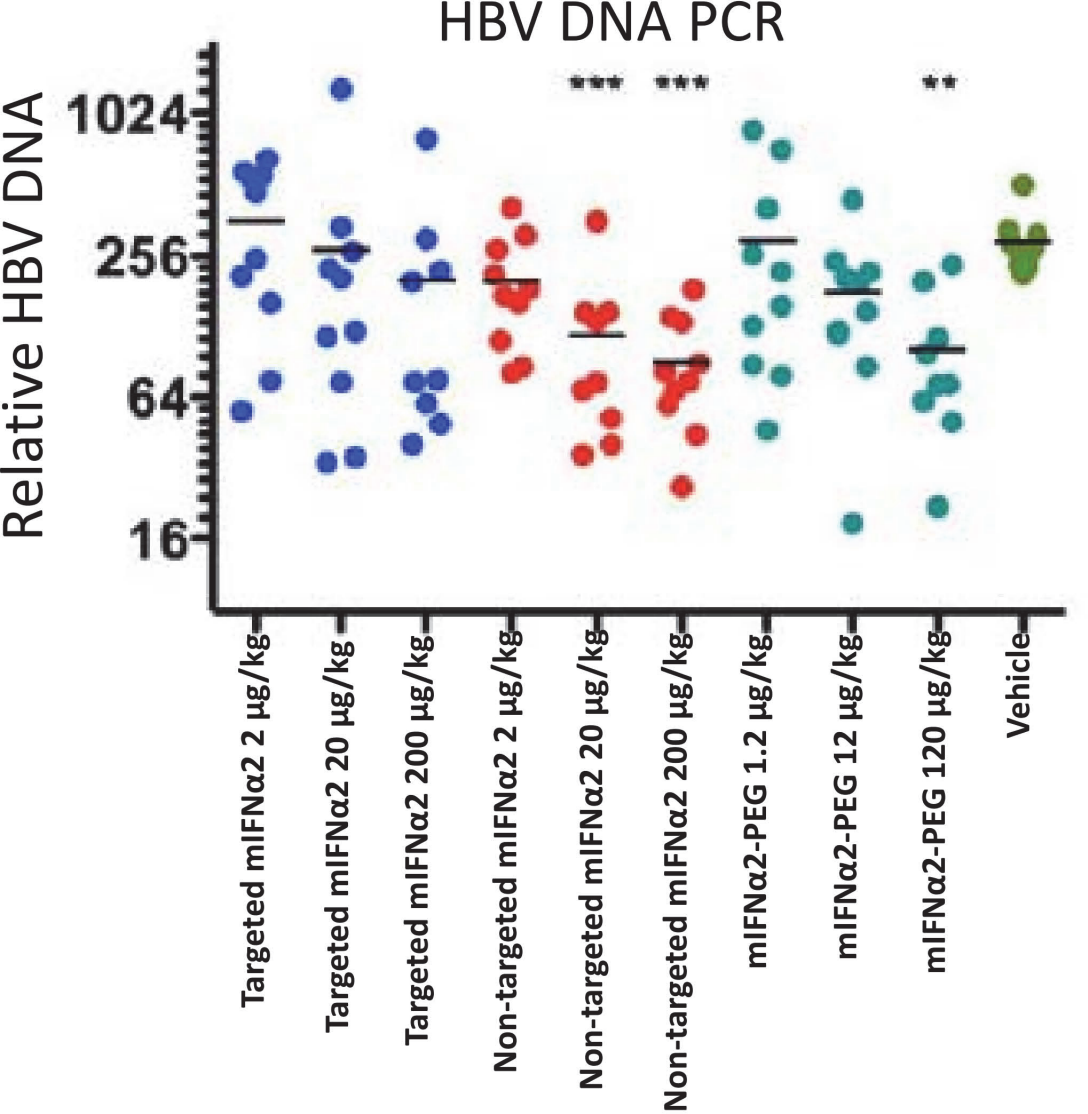
Antiviral efficacy of mIFNα2-dAb fusions and PEGylated mIFNα2 following intravenous administration. Effect of single-dose liver-targeted IFN-α on liver HBV DNA using Southern blot hybridization (upper panel) and quantitative PCR (lower panel) in HBV transgenic mice. For statistical analysis, the data were transformed to natural log for one-way analysis of variance, after which Bonferroni’s comparison analysis was performed. (*P < 0.05, **P < 0.01, ***P < 0.001 compared to vehicle control values).

All three forms of interferon; non-targeted mIFNα2, targeted mIFNα2 and PEGylated mIFNα2 (mIFNα2-PEG) were efficacious in reducing HBV DNA in livers of transgenic mice when assayed 1 day after one intravenous injection, although the non-targeted mIFNα2 appeared to have the greatest reduction under these experimental conditions as compared to the other two forms using both Southern blot hybridization (Fig. 8, upper panel) or quantitative PCR analysis (Fig. 8, lower panel). PEGylated mIFNα2 has the lowest efficacy of the three study compounds in this study.

## Discussion

Targeting of therapeutic payloads to specific cells or tissues is an attractive concept in terms of improving the safety and efficacy of the therapeutic. We have previously shown that ASGPR specific dAbs can be fused to therapeutic payloads without affecting *in vitro* potency or affinity of either fusion partner and used to increase liver specific uptake as determined in microSPECT/CT studies [26]. Here, we demonstrate that domain antibodies specific to ASGPR expressed exclusively on hepatocytes can be used to modulate the pharmacokinetics of therapeutic proteins to which they are fused, but result in slightly reduced *in vivo* efficacy.

Following intravenous administration of a large bolus dose of mIFNα2-dAb fusions and measurement of compound levels in serum using antibody based capture and detection methods, we observed a reduction in the AUC _(0-∞)_ for targeted mIFNα2 compared to non-targeted mIFNα2 and an increased volume of distribution. However, in this study, half-life values were reasonably comparable. During the initial distribution phase, targeted mIFNα2 was found to have consistently lower concentrations in the serum than non-targeted mIFNα2. At 12–24 hours, the serum concentrations were comparable and from 24 hours onwards, targeted mIFNα2 serum levels became higher than non-targeted mIFNα2. After 24 hours, the elimination of both molecules is visually comparable, as represented by their similar half-life values. These results are consistent with reduced systemic exposure as a direct consequence of liver targeting via the ASGPR dAb DOM26h-196-61. This would account for the reduced serum concentrations in the initial distribution phase, whereas the increased serum concentration, compared to the non-targeted molecule, could be ascribed to dissociation of the targeted compound following uptake in liver and redistribution in blood, a so-called ‘depot’ effect.

In order to determine whether the apparent differences in serum pharmacokinetics would be maintained using an alternative route of administration, we administered subcutaneous doses of mIFNα2-dAb fusions and measured compound levels in serum using the same antibody based capture and detection methods. While some pharmacokinetic characteristics of liver targeting molecules appeared consistent with observations from the previous intravenous study, others did not. In this study, Cmax for targeted mIFNα2 in serum was significantly lower than for non-targeted mIFNα2, AUC_(0-∞)_, for targeted mIFNα2 was much lower than for non-targeted mIFNα2 and the volume of distribution, of the fraction absorbed, was apparently larger for targeted mIFNα2. All these points were, therefore, consistent with previous findings after intravenous administration. However, the terminal half-life values were longer in this study for non-targeted mIFNα2 than for the targeted molecule targeted mIFNα2, which had not been observed previously. However, this may be a result of the difference in data points used to fit the data during non-compartmental analysis. Taken together we suggest that one of the major perceived benefits of liver-targeting (reduced systemic exposure as a direct consequence of liver binding) is observed with multiple routes of administration, and that this is a characteristic of the targeted molecule, rather than the route of administration.

In order to determine whether the observed differences between targeted and non-targeted mIFNα2-dAb fusions would be observed at more ‘clinically relevant’ doses, we employed a more sensitive means of detection, namely gamma counting of ^111^In-DOTA conjugated molecules in blood and tissues. The overall conclusion from these studies was that, once again, the pharmacokinetic characteristics of the liver targeted mIFNα2 appeared to be different to those of the non-targeted mIFNα2, and that generally, these were in line with what we had seen in previous studies at higher doses, and were therefore indicative of liver targeting. However, while differences were observed, it did seem as though the magnitude of the differences between non-targeted mIFNα2 and targeted mIFNα2 were not as great as seen previously, perhaps as a result of the lower dose used.

The key differences observed between the molecules included lower Cmax values for targeted mIFNα2 compared to non-targeted mIFNα2 in blood and kidney, but higher for targeted mIFNα2 in the liver, a decrease in Tmax in the liver for targeted mIFNα2, a longer half-life value for targeted mIFNα2 in blood, a reduction in the AUC_(0-∞)_ for targeted mIFNα2 in the kidney compared to non-targeted mIFNα2, and a larger volume of distribution. These findings were consistent with previous studies [26] and were a trend to expect for a molecule designed to target the liver. However, the half-life values of targeted mIFNα2 in the tissues were found to be lower or comparable to those of non-targeted mIFNα2, which did not fit with the results obtained in blood. In addition the AUC _(0-∞)_ values in blood and liver were fairly comparable between molecules, when previously we had observed larger differences. However, when partial AUC values of 0–12 hours and 0–24 hours were calculated during non-compartmental analysis, differences between targeted mIFNα2 and non-targeted mIFNα2 became more apparent and were approximately 2-fold lower in blood for targeted mIFNα2 compared to non-targeted mIFNα2 while approximately 2-fold higher in liver. The decrease in the AUC _(0-∞)_ in the kidney observed with targeted mIFNα2 implied a lower exposure to non-target tissue types, while the reduction in Tmax suggested that the loading of the liver with targeted mIFNα2 occurs at a faster rate, presumably due to its anti-ASGPR activity.

We subsequently investigated whether reducing exposure of mIFNα2-dAb fusions in non-target tissues, whilst increasing it in the target organ, would result in modulation of gene expression profiles *in vivo*. By measuring expression of a panel of IFN-inducible genes by TaqMan, we have shown that the reduced systemic exposure of targeted mIFNα2 does indeed correlate with reduced induction of IFN-responsive expression in blood, which may have implications for modifying the safety profile of systemically administered compounds. However the same effect was observed in liver, with the targeted compound inducing lower levels of gene expression than the non-targeted compound.

Finally, we conducted a study to assess the efficacy of mIFNα2-dAb fusions and PEGylated mIFNα2 in reducing hepatic levels of HBV DNA. Both non-targeted mIFNα2 and targeted mIFNα2 show superior efficacy compared to PEGylated mIFNα2, which would be expected based on published data showing that *in vitro* potency of interferon alpha is significantly reduced by attachment of PEG.

By directly comparing the antiviral activity of non-targeted mIFNα2 and targeted mIFNα2, the potential benefit, in terms of clinical efficacy, of targeting type-I interferons to the site of viral infection in the liver can be determined. The results of this study show that the targeted targeted mIFNα2 is less efficacious than the non-targeted non-targeted mIFNα2. This is somewhat unexpected, given the results of pharmacokinetic, biodistribution and micro SPECT/CT pre-clinical imaging studies. Given that both compounds have comparable *in vitro* potency in cell based assays an increased local concentration of interferon alpha, as observed in mice administered targeted mIFNα2, would be expected to result in reduced levels of HBV DNA in comparison to mice administered the non-targeted non-targeted mIFNα2.

The reduced efficacy of targeted mIFNα2 compared to that of non-targeted mIFNα2 in this study may be due to internalisation of the targeted compound following cell surface binding to ASGPR, resulting in reduced ability of targeted mIFNα2 to bind cell surface interferon-receptors IFNAR1 and/or IFNAR2, thereby reducing the efficiency of type I interferon signalling through the JAK/STAT pathway. Alternatively, the lack of improved efficacy may be in part due to the inability of interferon alpha-ASGPR dAb fusion proteins to simultaneously engage both ASGPR and interferon receptors at the cell surface, and sequestration of targeted mIFNα2 to the high copy number ASGPR at the cell surface may limit interaction with the interferon receptor chains IFNAR1 and/or IFNAR2, which would result in negative impact on the activation of type I interferon signalling pathways.

It should be noted, however, that despite the failure to improve the efficacy of interferon alpha in this pre-clinical model by liver-targeting via ASGPR binding, a significant reduction in the level of HBV DNA was still observed in animals injected with targeted mIFNα2 compared to animals receiving injections of vehicle only. This result, coupled with the results of pharmacokinetic, biodistribution and pre-clinical imaging studies referred to above, demonstrates conclusively that liver-targeting of therapeutically relevant payloads using ASGPR binding dAbs can be used to develop molecules which retain some *in vivo* activity (though activity appears to be lower than that observed with the non-targeted control), but show reduced exposure in non-hepatic tissues and blood. Such molecules could have potential benefit in treating disease indications for which current therapies, although efficacious, are limited in their utility by activity in off-target tissues. The potential of the liver-targeting dAb platform for improving the *in vivo* efficacy of alternative therapeutically relevant payloads waits further investigation.
