# Correction: Pharmacokinetic Characteristics, Pharmacodynamic Effect and *In Vivo* Antiviral Efficacy of Liver-Targeted Interferon Alpha

**DOI:** 10.1371/journal.pone.0124463

**Published:** 2015-04-10

**Authors:** The PLOS ONE Staff

There is an error in Fig 8. The upper panel of the figure was omitted. Please see the corrected Fig 8 here.

**Fig 8:**
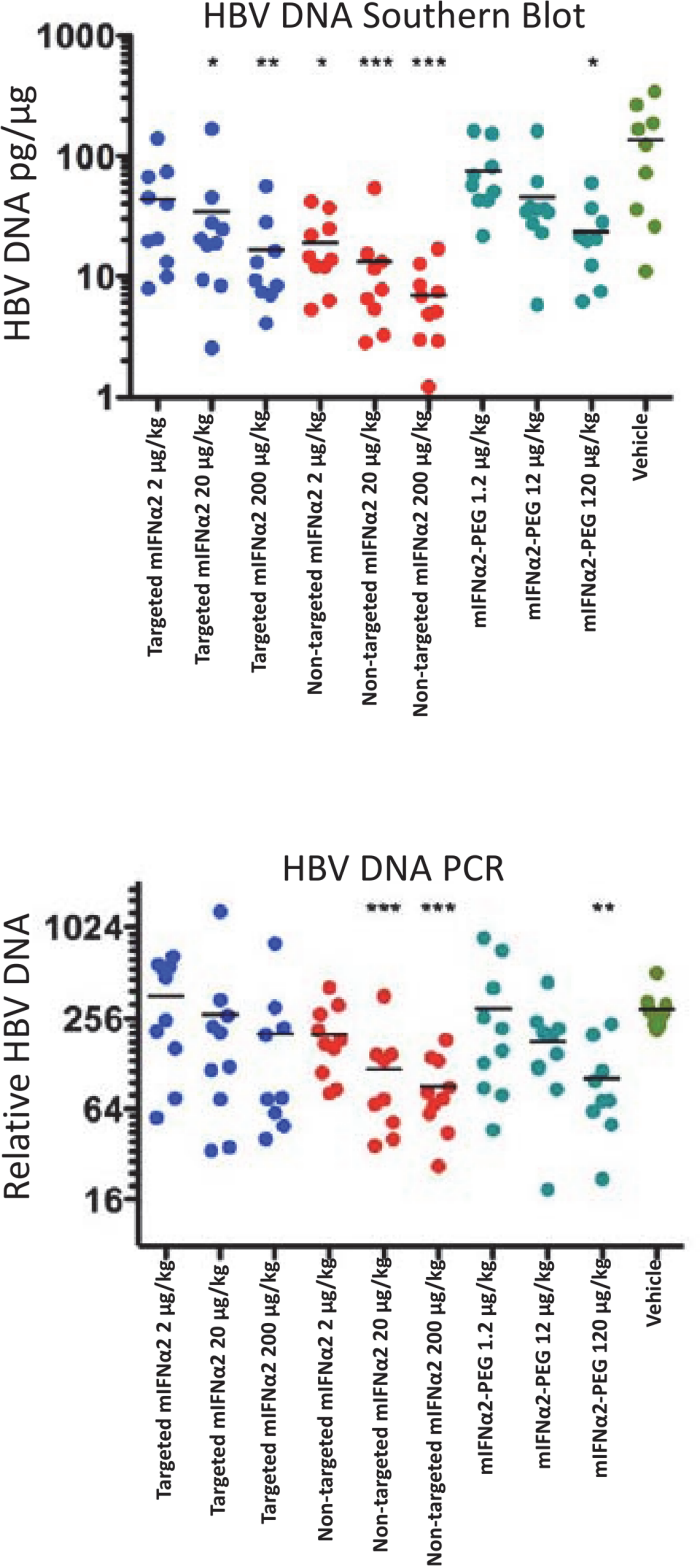
Antiviral efficacy of mIFNα2-dAb fusions and PEGylated mIFNα2 following intravenous administration. Effect of single-dose liver-targeted IFN-α on liver HBV DNA using Southern blot hybridization (upper panel) and quantitative PCR (lower panel) in HBV transgenic mice. For statistical analysis, the data were transformed to natural log for one-way analysis of variance, after which Bonferroni’s comparison analysis was performed. (*P < 0.05, **P < 0.01, ***P < 0.001 compared to vehicle control values).
